# Effectiveness of Manual Therapy and Acupuncture in Tension-Type Headache: A Systematic Review

**DOI:** 10.7759/cureus.17601

**Published:** 2021-08-31

**Authors:** Arifa Turkistani, Arpita Shah, Arunima Mariya Jose, Joao Pedro Melo, Kanita Luenam, Patricia Ananias, Sayma Yaqub, Lubna Mohammed

**Affiliations:** 1 Internal Medicine/Family Medicine, California Institute of Behavioral Neurosciences & Psychology, Fairfield, USA; 2 Family Medicine, California Institute of Behavioral Neurosciences & Psychology, Fairfield, USA; 3 Internal Medicine, Sree Gokulam Medical College and Research Foundation, Thiruvananthapuram, IND; 4 Psychology, California Institute of Behavioral Neurosciences & Psychology, Fairfield, USA; 5 Pathology, California Institute of Behavioral Neurosciences & Psychology, Fairfield, USA; 6 Public Health, The University of Texas Health Science Center at Houston, Houston, USA; 7 Internal Medicine, California Institute of Behavioral Neurosciences & Psychology, Fairfield, USA

**Keywords:** chronic headache, non-pharmacologic treatment, chronic daily headache, tension-type headache, manual therapy, acupuncture

## Abstract

Tension-type headache is one of the most prevalent types of headache. The common presentation is a mild-to-moderate dull aching pain around the temporal region, like a tight band around the forehead, neck, shoulder, and sometimes behind eyes. It can occur at any age but most commonly in the adult female population. The exact underlying mechanism is not clear but muscle tension is one of the main causes, which can be due to stress and anxiety. There are several non-pharmacologic treatment options suggested for tension-type headaches, such as cognitive behavioral therapy, relaxation, biofeedback, acupuncture, exercise, manual therapy, and even some home remedies. This systematic review was performed to evaluate the effectiveness of acupuncture and manual therapy in tension-type headaches. The literature search was primarily done on PubMed. Eight articles involving 3846 participants showed evidence that acupuncture and manual therapy can be valuable non-pharmacological treatment options for tension-type headaches. Acupuncture was compared to routine care or sham intervention. Acupuncture was not found to be superior to physiotherapy, exercise, and massage therapy. Randomized controlled trials done in various countries showed manual therapy also significantly decreased headache intensity. Manual therapy has an efficacy that equals prophylactic medication and tricyclic antidepressants in treating tension-type headaches. The available data suggests that both acupuncture and manual therapy have beneficial effects on treating symptoms of tension-type headache. However, further clinical trials looking at long-term benefits and risks are needed.

## Introduction and background

**Figure 1:**
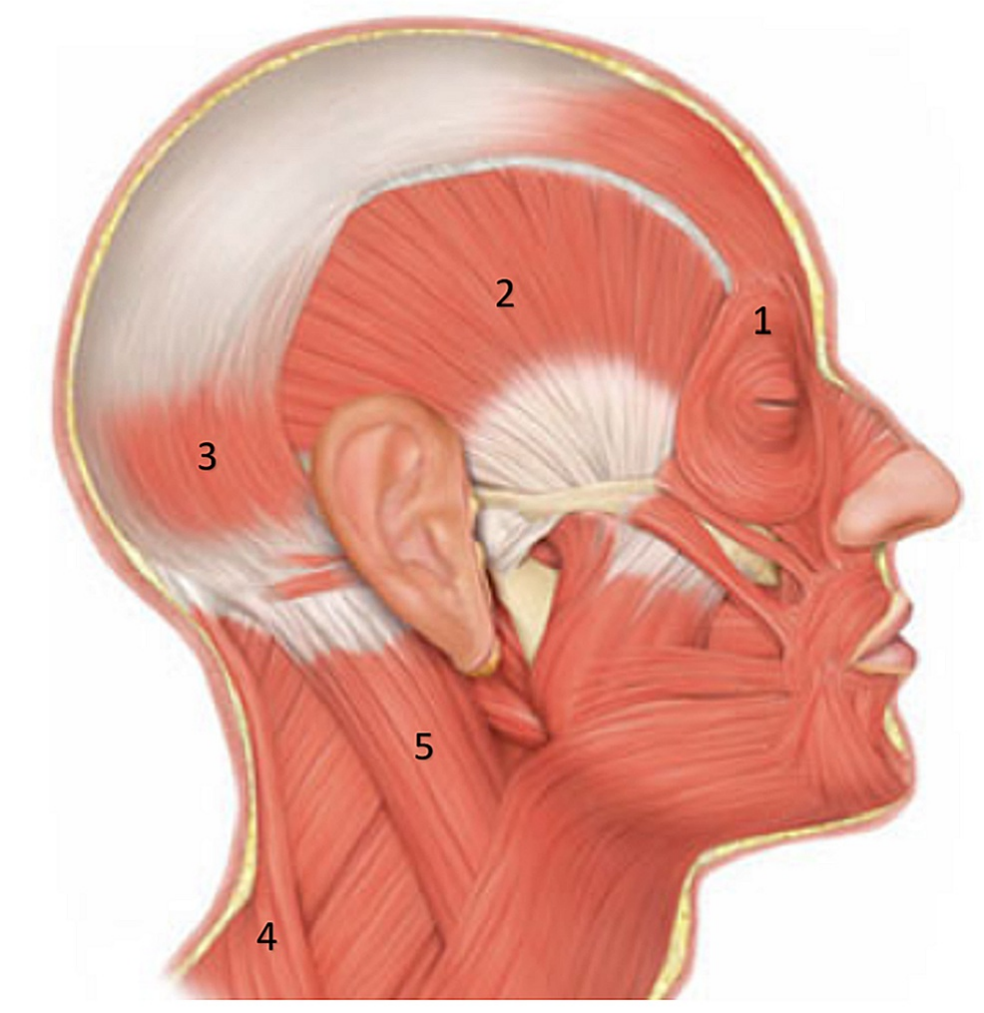
Areas involved in tension type-headache are (1) upper eyes, (2) ears, (3) occipital, (4) trapezius, (5) sternocleidomastoid The image is adapted from Muscles of the Head: http://www.musclesused.com/muscles-facial-expression/ (Public domain).

Tension-type headaches (TTH) are one of the most common complaints seen by family practitioners. The estimated lifetime prevalence of headaches is 66%, with tension-type headaches accounting for 36-78% of these. Approximately 90% of people have experienced headaches at least once in their lifetime and 14% of adults have TTH once per week [1-3]. This makes them the most common type of primary headache and the second most prevalent chronic condition. The characteristic description of TTH is dull, pressing, or tight headaches which are mild-to-moderate intensity [4]. They are also typically bilateral, diffuse, non-pulsatile, constant, tightening quality, and unmodified by physical activity [5]. The most common areas involved in TTH are shown in Figure 1. Due to vague symptoms, diagnosis of TTH can be challenging and usually is done by excluding all other causes of headache [6]. Associated symptoms may include photophobia and phonophobia [7]. They can have significant effects on quality of life and can interfere with daily activities even sometimes cause disability at work or school leading to socioeconomic burden [8]. Although they are so pervasive, the exact etiology and pathophysiology of TTH have been unclear, and treatment options have been vague [4,7].

Treatment for TTH includes pharmacologic and non-pharmacologic options [9]. Pharmacologic treatments mainly include analgesics, anticonvulsants, antidepressants, non-steroidal antiinflammatory drugs (NSAIDs), and triptans [10]. Non-pharmacologic treatments can be used alone or as an adjunct to pharmacologic therapy. These include cognitive-behavioral therapy (CBT), biofeedback, self-management therapies such as relaxation or mindfulness training, acupuncture, nutritional supplementation (magnesium, vitamin B12, vitamin B6, and coenzyme Q10), and physical therapies [11]. These non-pharmacologic therapies have been seen to be as effective or better than usual care in reducing headache intensity as well as in improving quality of life [9]. Several non-pharmacologic therapies have also been useful for TTH prevention [9,11].

Physical therapies have been reported as some to be the most frequently used alternative or complementary treatments for headaches [11]. One of the most common forms of physical therapy is known as manual therapy (MT) [11,12]. One proposed mechanism for the effect of manual therapies is the increase in pressure-pain thresholds with successive treatments on myofascial trigger points [13]. Massages can provide better results when combined with cervical spine manipulation [14]. One of the adverse effects that occur in almost 50% of individuals after receiving manipulative therapy is muscle stiffness and soreness [15].

Acupuncture is a widely used alternative therapy that is well tolerated and has fewer side effects compared to drugs used in pharmacologic treatment [16,17]. In classic acupuncture, needles are inserted at selected points known as acupoints followed by manipulation (heat, physical force, or electrical stimuli) [18]. It is believed (in traditional Chinese medicine) that imbalances in energy flow cause illness, while fine needle insertion at acupoints correct these imbalances and restore harmony [19]. It is used in the treatment of many conditions, including pain management and particularly headaches. Its efficacy has been observed primarily in migraines and TTH [7]. Studies have suggested acupuncture reduces the need for medication, improves relaxation, promotes the release of endogenous opioids, and has physical and psychological homeostatic effects [16]. Advantages to acupuncture include effectiveness, favorable safety profile, and few adverse effects as a non-drug therapy [4,17]. Despite existing studies, the effectiveness of acupuncture for the prevention of TTH has remained controversial due to inconsistency in findings, preventing the widespread implementation of acupuncture for TTH [4,7].

TTH is a ubiquitous medical condition seen by primary care physicians. There are several non-pharmacologic treatment options including manual therapy and acupuncture which have suggested promising results, but widespread incorporation of these techniques has been limited by inconsistent findings on effectiveness [4,7,20]. In this systematic review, we aim to evaluate the efficacy of manual therapy and acupuncture for TTH, the prevalence of their use, and the efficacy of acupuncture compared to that of manual therapy.

## Review

Methods

The primary objective of this study was to judge the effectiveness of the non-pharmacological treatments acupuncture and manual therapy in relieving symptoms of tension-type headaches.

This literature review was performed on April 29, 2021, and followed Preferred Reporting Items for Systematic Review and Meta-Analysis (PRISMA) 2009 checklist guidelines. Types of studies included were observational studies, case reports, systematic reviews, and randomized control trials aimed to evaluate the effectiveness of treating TTH with acupuncture and manual therapy. Two authors independently screened all abstracts and excluded those that were irrelevant to the scope of this study, for example, studies focusing on other types of headaches such as migraine and cluster headache. Inclusion criteria included studies published within the last 10 years from 2011 to 2021, free full-text articles, language was restricted to English, and studies done on adult humans aged 19 years and older. Studies including headaches caused by other medical conditions, pregnant and lactating females suffering from headaches, headaches in the pediatric population were excluded.

**Figure 2:**
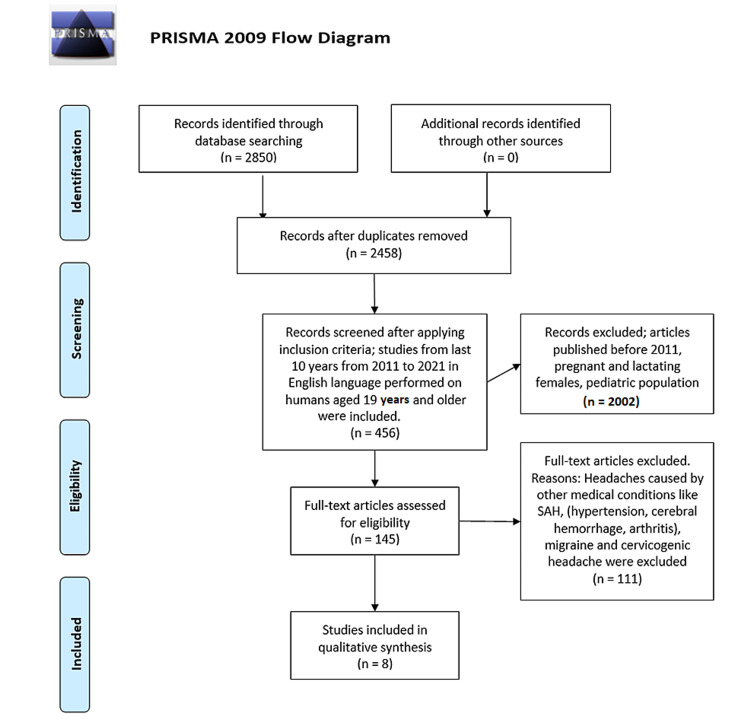
PRISMA flow diagram PRISMA: Preferred Reporting Items for Systematic Review and Meta-Analysis

We identified studies by a comprehensive PubMed search, duplicates were removed, and inclusion-exclusion criteria were applied. The summary of the number of results using keywords and medical subject heading (MeSH) terms is shown in Tables 1, 2. The selection of articles and quality assessment was performed by two authors using the New Castle-Ottawa Scale for the included observational studies and Cochrane risk of bias tool for randomized control trials. The results of selected studies were assessed and studies that showed the best evidence synthesis were included. Disagreements were solved by discussion. The summary of this process can be seen in the PRISMA flowchart in Figure 2.

**Table 1:** PubMed search results using keywords

Keyword	Database	Total number of results after inclusion/exclusion criteria
Chronic daily headache	PubMed	16,163 results
Tension-type headache	PubMed	1434 result
Acupuncture	PubMed	54 results
Chronic headache and tension-type headache	PubMed	1197 results
Manual therapy	PubMed	410 results
Nonpharmacological treatment	PubMed	104 results

**Table 2:** PubMed search results using MeSH terms MeSH: medical subject heading

MeSH Terms	Database	Total number of results after inclusion/exclusion criteria
"Tension-Type Headache/prevention and control"	PubMed	87 results
"Tension-Type Headache/rehabilitation"	PubMed	15 results
“Acupuncture” AND “headache, tension”	PubMed	2 results
“Manual therapy” AND “headache, tension”	PubMed	2 results

Results

This literature review included case reports, case-control trials, randomized controlled trials (RCTs), and systematic reviews. The key findings of all articles were collected and evaluated. The total number of participants included was 3926. A summary is provided in Table 3. All the relevant data was collected, analyzed, and divided into two categories: effectiveness of acupuncture and effectiveness of manual therapy.

**Table 3:** Findings of studies selected for analysis TTH: tension-type headache; MT: manual therapy; PPT: pressure-pain threshold; TENS: transcutaneous electrical nerve stimulation; MTrPs: myofascial trigger points; RCTs: randomized controlled trials; RRR: relative risk reduction; AT: autogenic training Headache index = frequency x intensity

Reference	Study type	Sample size (n)	Length of follow-up	Methods	Outcomes
Álvarez-Melcón et al. [20]	RCT	152	3 months	Relaxation technique (Schultz’s Autogenic Training, AT) was used in one group while the other group received combined treatment of AT plus physical therapy (cervical spine kinesiotherapy and posture correction exercises). Follow-up occurred at 4 weeks and 3 months. Frequency, intensity, and duration of pain were noted.	There was a decrease in all parameters of pain in both groups but a decrease in the frequency and intensity ware more significant in the combined treatment group (p≤0.001).
Moraska et al. [21]	RCT	56	6 weeks	One group received massage therapy focused on MTrPs in cervical muscle, bilateral upper trapezius, and sub-occipital muscle, second group either received placebo (detuned ultrasound) sessions or was waitlisted. Headache pain, intensity, and duration were noted every day in a diary.	Post hoc test showed a significant decrease in headache frequency in the massage intervention group (p<0.0003) and placebo (p=0.013) in comparison to the baseline, but no significant improvement was noted in wait list group.
Chaibi and Russell [22]	Systematic review of RCTs	181	8 days to 9 months	(1) In one study headache was diagnosed by a neurologist and RCTs were conducted by a physiotherapist. Head and neck massage was compared to detuned ultrasound (control group). (2) In a second study, biofeedback treatment was compared to the group receiving physiotherapy plus TENS. (3) In the third study, the effectiveness of standardized physiotherapy that includes massage, relaxation, and stretching twice a week was compared to an observational group.	(1) Headache intensity significantly decreased within 24 hours of massage (p<0.05) compared to detuned ultrasound which had no significant effect on TTH intensity. Mean headache intensity was decreased up to 24% after massage and 3% after detuned ultrasound. (2) Biofeedback treatment reduced the headache index by 83% while physiotherapy plus TENS reduced it by 97%. (3) Headache days were statistically decreased in the physiotherapy group post-treatment compared to the observation group (p<0.001).
Moraska et al. [13]	Placebo-controlled RCT	62	12 weeks	Three groups were made, one received placebo treatment with sham ultrasound sessions, a wait-listed group rested quietly on massage table for 45 minutes. And a third group received massage focused on trigger point release for 45 mins in bilateral upper trapezius and sub-occipital muscles. PPT was measured at MTrPs with pressure algometer before and after treatments.	PPT increased in muscles tested (MTrPs) after massage but not in sham ultrasound and wait-listed groups.
Mayrink et al. [16]	Placebo-controlled, double-blinded RCT	34	60 days	True acupuncture was compared to sham acupuncture in two equally divided groups. Quality of life was assessed before and after the treatment (Brazilian version quality of life questionnaire SF-6D 2002).	True acupuncture group showed statistically significant effectiveness in the treatment of TTH compared to sham acupuncture (p=0.0350). The functional capacity and general state of health were also improved in the true acupuncture group (p=0.0020).
Moore et al. [11]	Systematic review of surveys	1010	N/A	Prevalence of MT by type of provider was reported in chronic TTH populations. Self-reported effectiveness of MT was reported in surveys.	Combining results across all professionals administering MT showed significant effects of MT on relieving TTH which ranged from 17.0 to 82.0% (mean 42.5%).
Castien et al. [23]	RCT	82	8 weeks	Manual therapy was compared to usual care by general practitioners. Patients were followed for 8 weeks. Primary outcome was a decrease in headache frequency. Secondary outcomes included severity of headache and cervical range of motion.	There was a statistically significant decrease in headache frequency in the manual therapy group after 8 weeks (-6.4 days, 95% confidence interval -8.3 to -4.5). Headache severity and cervical range of motion were also statistically significantly improved in the manual therapy group compared to the usual care.
Linde et al. [7]	Systematic review of RCTS	2349	3 to 6 months	True acupuncture was compared to routine care or sham acupuncture in adults with episodic or chronic TTH.	Participants receiving true acupuncture showed 50% reduction in headache frequency compared to 43% reduction in sham acupuncture(routine care ) group after the treatment showed RR =12.5 and 95% CI 2.1-3.0 in trial 1 whereas trial 2 showed RRR of 11 and 95% CI 3.7-35.0).

Acupuncture

Two large RCTs compared acupuncture to routine care (sham intervention). The primary limitations were both being unblinded. In both trials, participants received acupuncture for three months after randomization (waiting list condition), so it was only possible to assess short-term effects up to three months after the start of the treatment. Participants receiving acupuncture along with routine care experienced on average a 50% decrease in headache frequency compared to the group receiving routine care only. Trial 1 showed a relative risk reduction (RRR) of 2.5 (95% CI 2.1-3.0); trial 2 showed RRR 11 (95% CI 3.7-35) (moderate quality of evidence) [7].

Another RCT considered the use of acupuncture as an auxiliary analgesic treatment. The research group received “true” acupuncture, in which the recommended points of traditional Chinese medicine were used. The control group received “sham” acupuncture, in which the needles were inserted into a stick-on device at the same points as the true acupuncture group. Quality of life was evaluated before and after treatment with the Brazilian version of the quality-of-life questionnaire: SF-6D, 2002. Participants in the true acupuncture group for TTH showed statistically significant improvement in all domains and improvement in overall quality of life [16]. Another prospective, randomized, controlled study with 401 patients with complaints of chronic headache (mostly migraine) in the primary care network in England and Wales compared acupuncture (12 sessions in three months) with medication and routine care. The acupuncture group had a steeper improvement in symptom scale (acupuncture 34% improvement/control 16% improvement), 22 fewer days of headache, used 15% less medication and had 25% fewer visits to the doctor [16].

Manual Therapy

Although data are limited, the use of manual therapy appears to be the most common non-pharmacological treatment utilized for recurrent TTH. Several participants and clinic population studies provide data for the effectiveness of MT. For chiropractic therapy, patient self-reporting of partial to full headache relief ranged from 27.0% to 82.0% (mean: 45.0%). For massage therapy, patient reports on partial to full headache relief ranged from 33.0% to 64.5% (mean: 45.2%). For osteopathy and physiotherapy, one study reported effectiveness as 17% and 36%, respectively. Combining results across all MT professions showed significant effects of manual therapy on relieving tension-type headache which ranged from 17.0% to 82.0% (mean: 42.5%). One general population study showed self-reported effectiveness for chiropractic and physiotherapy at 25.6% and 25.1%, respectively, for those with primary chronic headache including TTH [11].

Another cross-over RCT conducted by Spanish physiotherapists reported a statistically significant decrease in headache intensity 24 hours after massage (p<0.05). Mean headache intensity was reduced 24% after head and neck massage while mean headache intensity was reduced 3% after detuned ultrasound (control group). RCT conducted in the United States reported both spinal connective tissue manipulation and cervical mobilization groups had statistically significant improvement: 38% vs. 54% and 48% vs. 86%, respectively, at three-month follow-up (control group received superficial heat and classic massage to the neck and upper back). An RCT conducted in Turkey reported headache days were statistically significantly reduced in the physiotherapy group post-treatment (massage, relaxation, and stretching twice a week for four weeks) and at follow-up as compared to the observation group (p<0.001). Fifty-four percent of participants responded with >50% reduction in headache days. An RCT conducted in the Netherlands also reported a significant reduction in the frequency of headache after the use of physiotherapy consisting of mobilization of cervical and thoracic columns and exercise and postural correction, for nine sessions of 30 minutes each. At post-treatment and follow-up, the headache frequency was reduced 77% and 77%, respectively, in the manual therapy groups as compared to 23% and 35%, respectively, in the usual care groups who received reassurance, advice, lifestyle changes, and prescription of analytics and NSAIDs if necessary, by general practitioners [21].

Discussion

Effectiveness of Acupuncture

**Figure 3:**
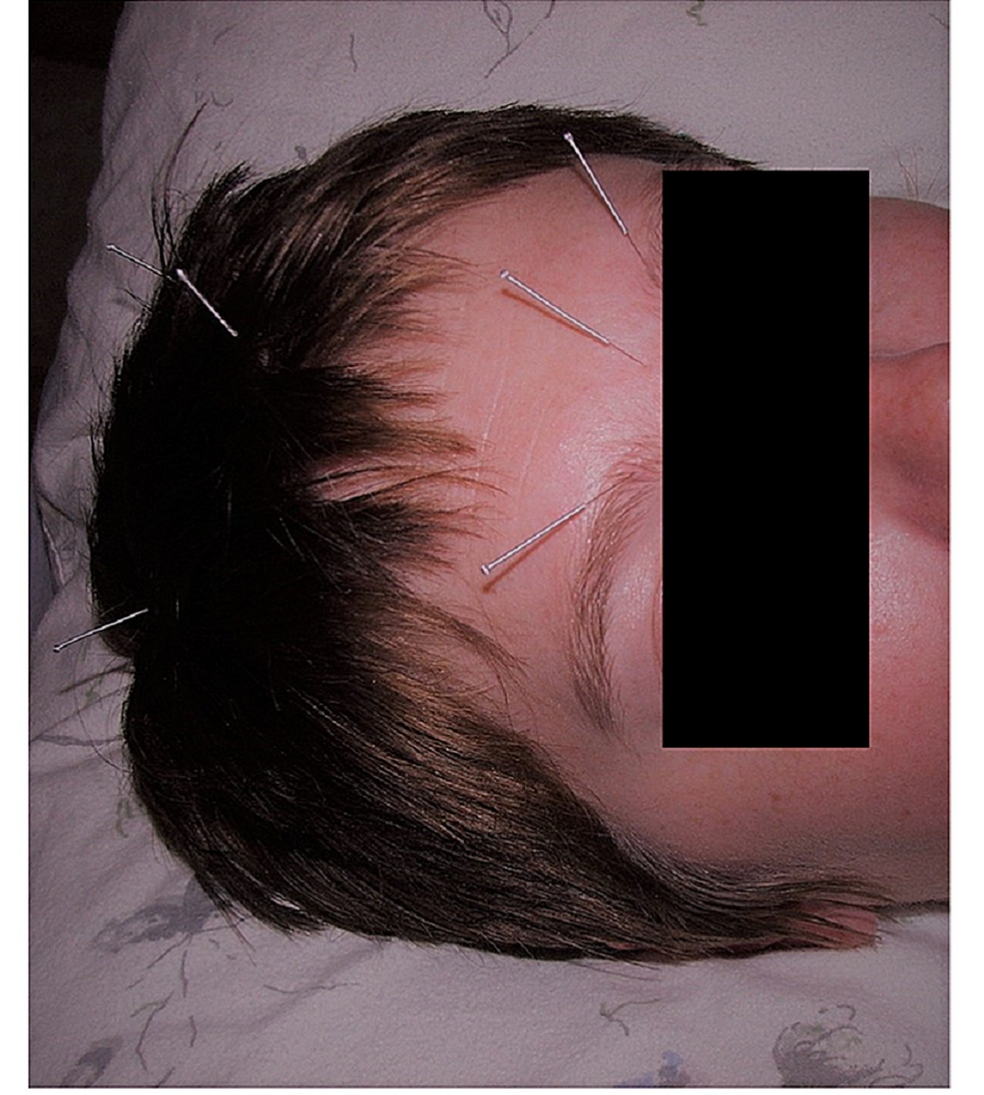
Example of common acupuncture points on the head The image is adapted from Facial acupuncture by Mscaprikell (2005): https://www.flickr.com/photos/mscaprikell/10972762/sizes/l/. CC BY-SA 2.0.

There have been many trials exploring the effectiveness of acupuncture in the treatment of TTH. Acupuncture involves the insertion of needles in specific acupoints for therapeutic effect (see example in Figure 3). Numerous acupoints could be targeted. The combination of acupoints used in a patient's treatment is called the prescription. Acupuncture theory guides the creation of prescriptions. There are 12 standard meridians (principal meridians) which are divided into yin and yang meridians, providing energy layers that protect the integrity of our body channels (including organs such as lung, heart kidney, liver, spleen). Yin meridians are present on inner surfaces and yang meridians are present on outer surfaces. They are paired together, for example, the large intestine, stomach, bladder, and gallbladder are paired. When yang meridians disturb the yin meridians, an imbalance is created that leads to chronic headaches. By stimulating “acupoints” of yang meridians a harmony can be restored that prevents disturbance in yin meridians. There are no standard prescriptions used in studies of acupuncture. The studies that were done until now used various prescriptions based on a combination of theory and practitioner experience rather than a standard. The development of a standard effective prescription would help to optimize treatment as well as reduce confounding from an evidence-based medicine perspective. The study by Lu et al. looked at the most prevalent and effective acupoints. The most used acupoints were Feng Chi (GB20), Beihai (GV20), Taeyang (EX-HN5), Hege (LI04), and Taichung (LR03), summarized in Table 4. They suggested a standard prescription for TTH based on a review of widely used and effective acupoints. Specifically, they suggest using points on the head (GB20, GV20, and EX-HN05) along with points on the limbs (LI04 and LR03). Future studies using this prescription could result in higher quality studies on the effectiveness of acupuncture [24].

**Table 4:** Top five most used acupoints in treatment of tension-type headache

Acupoint	Frequency	Body parts involved
Fengchi (GB20)	100	Face
Baihui (GV20)	74	Head, face, and neck
Taiyang (EX-HN5)	68	Head, face, and neck
Hegu (LI04)	49	Upper limb
Taichong (LR03)	47	Lower limb

A review from the Cochrane Institute showed that acupuncture in migraine is equally as effective or even more effective than preventive medication and has fewer adverse effects. In TTH, acupuncture did not seem superior to physiotherapy, massages, or exercises; however, new studies were suggested to rectify the methodological flaws. The authors conclude that acupuncture could be a valuable nonpharmacological tool in patients with episodic or frequent chronic headache crises [16].

There are several theories regarding the mechanism of TTH. One that connects to acupuncture theory describes the role of muscle nociceptors, myofascial trigger point tenderness, and muscle contraction in TTH. Myofascial trigger points are distributed along with the GB, GV, and EX-HN05 meridians on the head and neck. Stimulating these points can provide direct relief from TTH. Fengchi (GB20) and Baihui (GV20) are the core acupoint combinations with beneficial effects. Acupuncturing GB20 is thought to regulate blood flow, have antiinflammatory properties, and increase mast cells and macrophages which further inhibits hyperalgesia. It also is thought to affect the electromyographic activity of the sternocleidomastoid and trapezius muscle to improve pain tolerance. GV20 stimulation is thought to relieve cerebral vasospasm to further relieve TTH symptoms. The combination of GB20 and GV20 is the most used set of acupoints as they are thought to provide rapid response and symptomatic relief [9]. One RCT from Germany also found the points GB20, LR03, and LI04 to be high-frequency points used in 96%, 97%, and 67% of treatments sessions, respectively [20].

A recent RCT looking at acupuncture regimens including 20 sessions over four weeks found that the therapeutic effect was maintained for 20 weeks. Another systematic review showed a placebo effect related to "sham" acupuncture which lasted up to 12 weeks. There may be a short-term benefit related to the placebo effect, however, acupuncture seems to have a longer-term beneficial effect that extends beyond placebo [25].

TTH is quite common in young adults, especially at college-going age. A randomized controlled trial was done on 152 university students (selection was done according to the international headache society) as the prevalence of TTH is high in colleges and universities. Eighty-four were women (55.3%) and 68 were men (44.7%). The mean age was 20.42 years. Two groups were made, and one was treated with acupuncture only while the other was treated with a combination of acupuncture, spine kinesiotherapy, and posture training. Both groups showed an effective decrease in TTH but there was a significantly larger decrease in frequency and intensity of pain in the group receiving combined therapy (p<0.01) [20].

Effectiveness of Manual Therapy

Manual therapy, although limited by factors such as funding and regional availability, still appears to be substantially prevalent. Physiotherapy may be more prevalent in parts of Europe and chiropractic treatments may be more prevalent in Australia and the United States. Overall, when looking at different modalities of MT together, it is likely the most common type of non-pharmacologic therapy utilized for headaches in many countries [11].

One theory behind the effectiveness of MT involves myofascial trigger release points (MTrPs). Episodic (ETTH) and chronic (CTTH) tension-type headache populations were found to have an increased number of MTrPs in pericranial musculature that reproduced the headache complaint when stimulated, similar to the light pinching technique used in acupuncture. It was hypothesized that massage of MTrPs in the cervical musculature would result in a reduction in headache pain. Moraska et al. suggested that increase in the pressure-pain threshold (PPT) at MTrPs is a mechanism underlying the beneficial effect of MT on headache frequency. They found massage therapy reduced MTrP PPT from beginning to end of the treatment phase (p<0.001) and that the change in PPT predicted a decrease in the average number of headaches from the baseline to the follow-up period (p<0.001). Although this was a significant effect, headache frequency also decreased in the placebo arm which did not impact trigger point tenderness, implying other mechanisms for headache reduction are also present [21]. In another RCT, myofascial trigger point release was used as part of manual therapy. Two groups of 20 participants were randomized to massage, placebo, and a waist list group. The post-hoc analysis identified that headache frequency decreased for both massage (p=0.0003) and placebo (p=0.013) relative to their baseline but not for the wait-list group (p=0.098). No statistical difference was detected between the massage and placebo treatment groups (p=0.26). At post-treatment, the vast majority of those in the massage condition reported improvement (84.7%), compared with 50% improvement in the placebo group and 0% in the wait-list group (p<0.001) [21].

Another RCT was done on 82 participants with CTTH. Participants were equally divided into two groups, one assigned to MT and the second to usual care by their general practitioner. The primary outcome was decreased headache frequency while secondary outcomes included severity of headache and cervical function. Both groups were followed and checked for eight weeks. A statistically significant decrease in headache frequency was noted in the MT group (95% confidence interval -8.3 to -4.5; effect size 1.6) compared to the usual care group. The severity of headache and cervical function also showed a significant difference in favor of MT at eight weeks [23].

Antidepressants (TCAs) have been used in the management of TTH. In one randomized control trial, manual therapy was compared to the prophylactic use of antidepressants. Fifty-four percent, 82%, and 85% of the participants in the three physiotherapy RCTs had >50% reduction in headache index (headache frequency x headache intensity) post manual therapy and the effect was maintained on six-month follow-up, compared to 40-70% of participants receiving antidepressant treatment also showed similar results but side effects from TCA were noted especially at six-month follow-up [22]. Thus, it is more beneficial to use manual therapy in treating TTH compared to antidepressants because it has no adverse effects compared to the latter, or risk of overuse.

The quality of studies looking at the effectiveness of manual therapies for TTH are low and comparison of results is limited due to differences in technique, similar to acupuncture. One primary limitation is the difficulty in establishing a double-blinded study. Thus, while patients seek manual therapies commonly for headache relief, providers need to remain mindful of the evidence and provide adequate informed consent to patients. More research using standardized protocols in manual therapies is required to assess the true short- and long-term benefits [11]. 

Adverse Effects of Acupuncture and Manual Therapy

Common adverse effects of acupuncture include bruises, pain, and localized edema at needle insertion sites. However, these are not associated with long-term effects. One of the most common side effects of acupuncture is micro bleeding which is defined as bleeding that took from 10 to 30 seconds to resolve. A retrospective study was conducted to assess the severity of micro bleeding, specifically in patients taking anticoagulants. A total of 316 patients were divided into three groups: group A taking a direct oral anticoagulant (dabigatran, rivaroxaban, apixaban); group B taking antiplatelet therapy (aspirin, clopidogrel, triflusal); and group C receiving no anticoagulants (control group). All groups received various sessions of acupuncture, totaling 10,177 sessions. Micro bleeding occurred at a rate of 3.9% in the oral anticoagulant group, 5.6% in the antiplatelet group, and 5.1% in the control group. There was no significant increase in rates of micro bleeding among patients taking oral anticoagulants or antiplatelets. There were also no serious side effects such as massive hemorrhage in any of the groups. Overall, acupuncture seems to be a safe treatment in patients taking anticoagulants with self-limited adverse effects [26]. However, this and other studies related to bleeding in acupuncture have been limited by a small sample size, and larger double-blinded studies are needed to further assess the safety profile of acupuncture.

Adverse effects of manual therapy muscle stiffness, soreness, increased pain, fatigue, and weakness. These have been reported in about 50% of adults after manual therapy but are also self-limited. Serious side effects leading to hospital admission or death include vertebrobasilar injury or dissection, hematoma, disk herniation, and fracture. The true incidence of these serious adverse events is likely rare based on prior studies; however, the ranges of reported frequencies have varied broadly from as much as one in 20,000 to as little as one in 1,000,000 [15].

Limitations

There are several limitations in this review. In the studies reviewed, pediatric populations were not included. Conducting an RCT on manual therapy is also both time and cost consuming, and blinding often is difficult as there is no standardized sham treatment to use as a control group. Thus, all of the included studies were pragmatic or used a control consisting of either no treatment or “routine” care. Several biases may also affect these results. Publication bias may have been a factor as unpublished and non-English RCTs were not included.

## Conclusions

Our review covers an integrative review on the prevalence and effectiveness of acupuncture and manual therapy for tension-type headaches. The available results suggest that acupuncture and manual therapy are effective for treating chronic tension-type headaches. Although evidence-based recommendations are limited by the availability of data, there do seem to be significant benefits to these therapies in the treatment of tension-type headache. Due to limitations related to lack of standardized studies and a paucity of data, the ability to make strong conclusions is limited. Thus, it is unclear the magnitude of the benefit of acupuncture or manual therapy when compared to routine care or even when compared to each other. However, these findings show the potential of acupuncture and manual therapy for tension-type headache and give suggestions for future research.
